# Role of Extrinsic Apoptotic Signaling Pathway during Definitive Erythropoiesis in Normal Patients and in Patients with β-Thalassemia

**DOI:** 10.3390/ijms21093325

**Published:** 2020-05-08

**Authors:** Olga Raducka-Jaszul, Dżamila M. Bogusławska, Natalia Jędruchniewicz, Aleksander F. Sikorski

**Affiliations:** 1 Department of Cytobiochemistry, Biotechnology Faculty, University of Wrocław, Joliot-Curie 14a, 50-383 Wrocław, Poland; olgaraducka@gmail.com; 2 Department of Biotechnology, Faculty of Biological Sciences, University of Zielona Góra, Szafrana 1, 65-516 Zielona Góra, Poland; d.boguslawska@wnb.uz.zgora.pl; 3 Research and Development Center, Regional Specialist Hospital, Kamieńskiego 73a, 51-154 Wroclaw, Poland; jedruchniewicz@wssk.wroc.pl

**Keywords:** apoptosis: apoptotic extrinsic pathway, erythropoiesis, β-thalassemia, Fas/FasL, TRAIL/TRAILR, death domains

## Abstract

Apoptosis is a process of programmed cell death which has an important role in tissue homeostasis and in the control of organism development. Here, we focus on information concerning the role of the extrinsic apoptotic pathway in the control of human erythropoiesis. We discuss the role of tumor necrosis factor α (TNFα), tumor necrosis factor ligand superfamily member 6 (FasL), tumor necrosis factor-related apoptosis-inducing (TRAIL) and caspases in normal erythroid maturation. We also attempt to initiate a discussion on the observations that mature erythrocytes contain most components of the receptor-dependent apoptotic pathway. Finally, we point to the role of the extrinsic apoptotic pathway in ineffective erythropoiesis of different types of β-thalassemia.

## 1. Introduction 

Erythrocytes have a life span of approximately 120 days, at the end of which they become senescent and are removed from the circulation. Premature removal of red blood cells occurs by eryptosis, a form of stress-induced, programmed cell death which leads to the removal of defective cells without the release of the cell content. During this process, changes similar to apoptosis, such as cell shrinkage, membrane blebbing (release of small membrane vesicles) and exposure of phosphatidylserine on the cell membrane surface can be observed [1,2]. Eryptosis is a strictly controlled process involving receptors, ion channels and a wide range of signaling molecules (mostly kinases) and mediators leading to the above-mentioned morphological changes of the erythrocyte followed by its phagocytosis by macrophages. One of the main events of eryptosis is an increase in cytosolic Ca^2+^ concentration, which is mainly a consequence of Ca^2+^ entry through prostaglandin E2 (PGE_2_)-activated and erythropoietin-inhibited non-selective cation channels [3,4]. An increased Ca^2+^ ion concentration activates the Gardos channel, which facilitates K^+^ efflux, resulting in cell dehydration and shrinkage [5]. Further consequences of elevated Ca^2+^ content are loss of membrane phospholipid asymmetry (“scrambling”) and calpain activation, which is responsible for proteolysis of cell membrane skeleton proteins and membrane loss via blebbing [6]. The scrambling of membrane phospholipid asymmetric distribution and cell shrinkage caused by Ca^2+^ is enhanced by ceramide arising from sphingomyelin hydrolysis, which is another stimulus of eryptosis. Another important activation mechanism of eryptosis is energy depletion, which has an impact on the replenishment of reduced glutathione (GSH), protein kinase C (PKC) activity and phosphorylation of membrane proteins. Eryptosis can also be stimulated by oxidative stress, which activates Ca^2+^-permeable cation channels, Cl^−^ channels and caspases. Additionally, eryptosis can be triggered by dysregulation of several kinases, e.g., Janus kinase 3 (JAK3), AMP-activated kinase (AMPK), p21-activated kinase 2 (PAK2) [5,6,7].

Erythropoiesis [8] is the process through which erythrocytes are produced and is governed by the balance between the rate of production and destruction of erythroid cells. Growing evidence indicates that an apoptotic mechanism is involved in the control of erythropoiesis. Two separate signaling pathways are involved in regulation of apoptosis during erythropoiesis: the mitochondrial dependent intrinsic pathway and death receptor dependent extrinsic pathway [9,10]. 

The intrinsic pathway, which is mitochondrial-dependent, works in response to death stimuli such as DNA damage, chemotherapeutic agents, serum starvation and UV radiation. The intrinsic apoptotic pathway is controlled by members of the B-cell lymphoma 2 (BCL2) protein family, which regulate mitochondrial outer membrane permeability to proteins such as cytochrome C (CytC). The BCl2 protein family is subdivided into three groups based on their structure and functions. The first group comprises multidomain pro-apoptotic BCL2 family proteins: BCL2 antagonist/killer (BAK) and BCL2-associated X protein (BAX). BAK and BAX contain three BCL2 homology (BH) domains and can directly permeabilize mitochondrial outer membrane. The second group includes multidomain anti-apoptotic BCL2 family proteins such as BCL2, BCL-X large (BCL-X_L_), BCL2-like protein 2 (BCLW), BCL-2 like protein 10 (BCLB), myeloid cell leukemia 1 (MCL1) and BCL2-related protein A1 (BFL1). These proteins have four BH domains and inhibit apoptosis by binding to pro-apoptotic BCL2 family proteins. The last group is called BH3-only proteins and contains BCL2 interacting mediator of cell death (BIM), p53 upregulated modulator of apoptosis (PUMA), BH3 interacting-domain death agonist (BID), BCL2 associated death promoter (BAD), Phorbol-12-myristate-13-acetate-induced protein 1 (NOXA), BCL2 interacting killer (BIK), BCL2 modifying factor (BMF) and activator of apoptosis harakiri (HRK) proteins. The BH3-only proteins have a single BH3 domain which is critical for the interaction with other BCL2 family proteins [11,12,13]. 

Studies mainly based on a mouse model showed that BCL-X_L_ protein is the most critical anti- apoptotic factor in erythropoiesis [14]. The expression level of BCL-X_L_ varies among the stages of erythropoiesis. In CD34^+^ cells there is a low level of BCL-X_L_, which then markedly increases from a burst-forming unit-erythroid (BFU-E) to colony-forming unit-erythroid (CFU-E) and to CFU-E/proerythroblasts (ProEs). The maximum level of BCL-X_L_ is reached at polychromatophilic erythroblast (PolyE) stages and falls at terminal stages of erythroid maturation. For comparison, the BCL2 protein was found to be present only at early stages of erythroid differentiation (stage from BFU-E to CFU-E and to CFU-E/ProEs) [9]. There is evidence that the overexpression of the BCL-X_L_ gene in mouse erythroblasts enables them to differentiate into mature erythrocytes in the absence of erythropoietin (Epo) [15]. Other factors which are involved in erythroid maturation include BAK [16], BAX [16], MCL1 [17,18], PUMA [17,19], BIM [19,20] and NOXA [21]. 

The extrinsic apoptotic pathway triggers apoptosis by the binding of ligands to death receptors, which leads to the formation of a death-inducing signaling complex (DISC) and, in consequence, caspases activation. These receptors are members of the tumor necrosis factor receptor superfamily (TNFRSF).

Extrinsic and intrinsic apoptotic pathways can converge in erythropoiesis. The main link between these pathways is made by the BH3-only protein BID, which is cleaved by caspase-8 [22] or caspase-10 [23]. During erythroid maturation, a characteristic shift from pro-apoptotic to an anti-apoptotic phenotype takes place. According to this concept, BID encoding gene expression is higher in early erythroblasts than in late stages [24]. BID is involved in the regulation of mitochondria depolarization and caspase-9 activation. There is evidence that the BID phosphorylation, and in consequence, its cleavage pattern, may determine whether erythroblasts will differentiate or undergo apoptosis. The authors suggest that, during erythroid differentiation, BID may be cleaved by caspase-10 on D38 residue generating truncated form, P18. Phosphorylation of this form by casein kinases 1 (CK1) leads to erythroblasts differentiation. In turn, inhibition of CK1 may induce apoptosis [25].

This review focuses on the impact of the extrinsic apoptotic pathway on normal human erythropoiesis. Similarities between eryptosis and apoptosis prompted studies aiming at the evaluation of whether erythrocytes possess apoptotic machinery similar to nucleated cells. Additionally, we discussed the influence of the extrinsic, receptor-dependent apoptotic pathway on ineffective erythropoiesis in patients with β-thalassemia.

## 2. Receptor-Dependent Apoptotic Pathway

**Figure 1:**
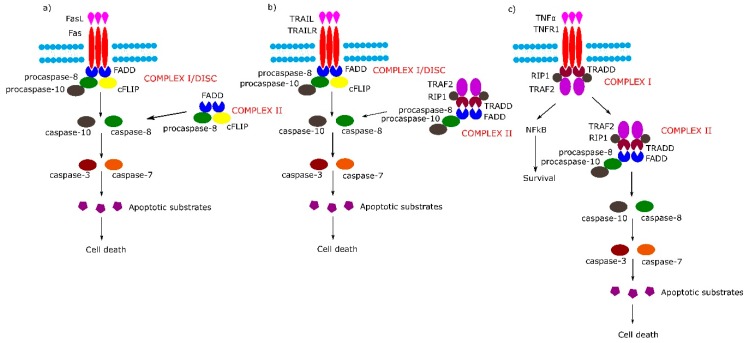
Extrinsic apoptotic signaling pathway. (**a**,**b**) Tumor necrosis factor (TNF) receptor superfamily member 6/tumor necrosis factor-related apoptosis-inducing (Fas/TRAIL) mediated apoptotic pathway leads to the formation of Complex I/death-inducing signaling complex (DISC) which consists of Fas-associated protein with death domain (FADD), procaspase 8/10 and cellular FLICE (FADD-like IL-1β-converting enzyme) inhibitory proteins (cFLIP). (**c**) Binding of tumor necrosis factor α (TNFα) to TNF-α receptor 1 (TNFR1) receptor causes the formation of Complex I (TNFR1-associated protein with death domain (TRADD), TNFR1, TNF receptor-associated factor 2 (TRAF2), TNFα-related receptor interacting protein (RIP), which in the next stage either activates the nuclear factor kappa-light-chain-enhancer of activated B cells (NF-κB) pathway or transforms into Complex II (TRADD, TNFR1, TRAF2, RIP, FADD, procaspase-8, procaspase-10).

The TNFRSF consists of TNF receptor-associated factor (TRAF)-interacting receptors, decoy receptors (DcRs) and death receptors (DRs). The DRs can further be classified according to the molecular mechanisms of apoptosis and necroptosis activation: TNF receptor superfamily member 6 (Fas), TNF-related apoptosis-inducing ligand receptor 1 (TRAILR1) and TNF-related apoptosis-inducing ligand receptor 2 (TRAILR2) belong to the first subgroup of the DRs, which on the cytosolic plasma membrane surface, directly interact with an adapter protein, Fas-associated protein with death domain (FADD) and tumor necrosis factor α (TNF-α) receptor 1 (TNFR1/p55), which belongs to the second subgroup of DRs that act directly with TNFR1-associated protein with death domain (TRADD) [26]. Schemes of mentioned above pathways are shown in Figure 1.

The DRs are characterized by an extracellular N-terminal domain with cysteine-rich domains (CRDs), a membrane-spanning region and a C-terminal, an intracellular domain which harbors a death domain (DD). While DRs share the CRDs with all members of the TNFRSF, the DD is only found in DRs. The CRDs contain the N-terminal pre-ligand assembly domain (PLAD) which is involved in receptor–receptor interactions and is involved in binding ligands of the TNF superfamily (TNFSF) [27,28]. The PLAD domains’ interactions cause spontaneous receptor dimerization and/or trimerization. In the case of CD95/Fas and TRAILR death receptors, PLAD–PLAD interaction has a high affinity, in contrast to the TNF1 receptor [29].

Death ligands exist in the cell membrane as trimeric type II transmembrane proteins belonging to the TNFSF. They contain a conserved C-terminal TNF homology domain (THD), an N-terminal intracellular proline-rich domain (PRD) and a stalk region. THD binds to CRDs of the TNFRSF. Membrane-bound death ligands can be processed in the stalk region by metalloproteases, which leads to the release of soluble molecules. The soluble death ligands also own THD and, in consequence, have the capacity to react with TNFRSF receptors [27].

According to the sequential model of TNFRSF receptor activation, a single TNFRSF receptor interacts with a TNFSF ligand trimer and forms a cell surface-bound TNFRSF receptor-TNFSF ligand_3_ complex. Next, in two subsequent stages, two additional monomeric TNFRSF receptors are added to create an active TNFSF ligand_3_–TNFRSF receptor_3_ cluster which is necessary for induction of death-inducing signaling complexes [29].

The binding of Fas with tumor necrosis factor ligand superfamily member 6 (FasL) leads to formation of the DISC, also termed complex I, consisting of FADD, procaspase-8, procaspase-10 and cellular FLICE (FADD-like IL-1β-converting enzyme) inhibitory proteins (c-FLIPs). The formation of DISC leads to activation of caspase-8, caspase-10 and in consequence to cleavage and activation of effector caspase-3 and caspase-7. Additionally, in some cell lines, a second cytosolic complex is formed upon ligand stimulation. This complex, called complex II, composed of FADD, procaspase-8 and c-FLIPs, might amplify caspase activation by processing caspase-3 (Figure 1a) [30].

Upon tumor necrosis factor-related apoptosis-inducing (TRAIL) stimulation, the TRAIL receptor forms membrane-associated DISC/complex I in a similar way to the Fas receptor and secondary complex, similarly to above-mentioned TNFR1 complex II (Figure 1b) [30].

Signaling through TNFR1 leads to the formation of complex I and then complex II. Complex I consists of TRADD, TNFR1, TNF receptor-associated factor 2 (TRAF2) and tumor necrosis factor α (TNFα)-related receptor interacting protein (RIP). Complex I assembles rapidly following TNF-α stimulation, and activates the nuclear factor kappa-light-chain-enhancer of activated B cells (NF-κB) signal transduction pathway, which activates survival signals. In the next step, complex I dissociates from the death receptor and binds FADD and procaspase-8 (complex II), the activation of which leads to an induction of apoptosis (Figure 1c) (reviewed in [30]).

**Figure 2:**
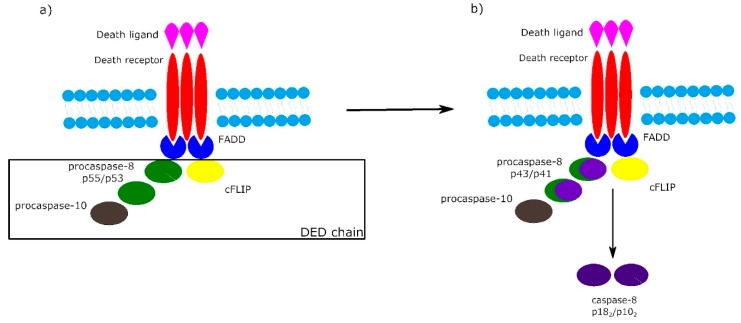
Death effector domain (DED) chains and procaspase-8 transprocessing. (**a**) Activation of procaspase-8 begins with DED chains, which consist of procaspase-8 p55/p53, procaspase-10 and c-FLIP. (**b**) Procaspase-8 homodimers are converted into active caspase-8 heterotetramers p18_2_/p10_2_ via the intermediate procaspase-8 p43/p41.

It is worth noting that the activation of procaspase-8 at DISC is driven by death effector domain (DED) “chains”, which include procaspase-8, procaspase-10 and c-FLIP. The number of procaspase-8 molecules in DED chains is 10 times higher than the number of procaspase-10 and c-FLIP molecules. The local increase of procaspase-8 concentration in DISC causes dimerization of procaspase-8. Then, procaspase-8 dimers (also called p55/p53) are converted by autocatalytic two-step transprocessing into mature heterotetrameric caspase-8 (p18_2_/p10_2_) while the DISC-bound caspase-8 prodomain can be replaced by a new procaspase-8 molecule. The termination of the DED chain’s elongation depends on its stability and association/dissociation rates of procaspase-8 to the chain (Figure 2) [26,31,32].

**Figure 3:**
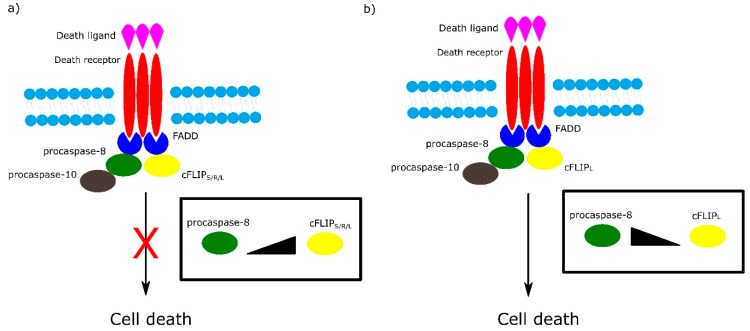
Role of different isoforms of cFLIP in extrinsic apoptotic pathway. The ratio of cFLIP to procaspase-8 in cFLIP-procaspases-8 heterodimer determines cell fate. (**a**) High levels of cFLIP_S/R/L_ block formation of procaspase-8 oligomer and in consequence inhibit cell death. (**b**) At low level of cFLIP_L_, cFLIP_L_ - procaspase-8 heterodimer activates cell death.

Among the most important regulators of the receptor-dependent apoptosis pathway are c-FLIP isoforms, which are major antiapoptotic proteins. Three isoforms can be distinguished as: short c-FLIP_S_, Raji c-FLIP_R_ and long c-FLIP_L_. All c-FLIP isoforms possess two N-terminal DED domains. c-FLIP_S_ and c-FLIP_R_ are truncated version of procaspase-8, whereas c-FLIP_L_ retains a full length procaspase-8 chain in which the catalytic cysteine residue within the active site is missing and thus has no proteolytic activity [32]. The isoforms of c-FLIP can heterodimerize with procaspase-8 via a co-operative and hierarchical binding mechanism. The result of c-FLIP_S_ or c-FLIP_R_ homo- or heterodimerization with procaspase-8 is prevention of DED-mediated caspase-8 oligomerization and inhibition of its activation. The ratio of c-FLIP_L_ to procaspase-8 in c-FLIP_L_-procaspase-8 heterodimers determines whether procaspase-8 will be activated or inhibited. At physiologically-low levels, c-FLIP_L_-procaspase-8 heterodimer activates procaspase-8. In contrast, high levels of c-FLIP_L_ block procaspase-8 oligomerization, which results in inhibition of caspase cascade activation (Figure 3) [32,33].

Other regulators of apoptosis are inhibitors of apoptosis (IAPs). The mammalian IAPs, X-linked IAP (XIAP), cellular inhibitor of apoptosis protein 1 (cIAP1) and cellular inhibitor of apoptosis protein 2 (cIAP2) contain three baculovirus IAP repeat (BIR) domains which mediate protein–protein interactions, UB-associated domain (UBA), which is responsible for interaction with ubiquitylated proteins, and the “really interesting new gene” (RING) domain conferring E3-ubiquitin ligase activity. Additionally, cIAP1 and cIAP2 have the caspase recruitment domain (CARD), which has the ability to inhibit their E3 ligase activity [34]. The BIR1 domain of XIAP binds the TGFβ-activated kinase 1 (TAK1)-binding protein 1 (TAB1) [35], whereas cIAPs’ BIR1 domain binds TRAF1 and TRAF2 [36]. In turn, the XIAP BIR2 domain is involved in inhibition of effector caspase-3 and caspase-7, while BIR3 binds to caspase-9 and prevents dimerization of this enzyme [37]. cIAP1 and cIAP2 can bind caspases through BIR2 and BIR3 domains, but do not inhibit them [38]. XIAP is able to induce ubiquitination of active caspases at the K48 residue, causing their (proteasomal) degradation. Additionally, XIAP is involved in covalent tagging of caspase-7 with ubiquitin-like protein NEDD8 leading to inactivation of this caspase [39]. cIAP1 and cIAP2 are able to ubiquinate caspase-3 and caspase-7 [40,41] but only cIAP1 targets them for proteasomal degradation [40].

The linear ubiquitin chain assembly complex (LUBAC) is another regulator of cell death. LUBAC is the only known E3 ligase complex for linear ubiquitination, which consists of ring finger protein 31 (RNF31, also known as HOIP), HOIL-1 and Shank-associated RH domain-interacting protein (sharpin) [42]. Recent studies showed that sharpin has a regulatory function in NF-κB and apoptotic signaling pathways. Absence of sharpin attenuates TNFα-mediated NF-κB activation [43,44,45]. Cleavage of RNF31 by caspase-3 and caspase-6 suppresses activity of LUBAC in the NF-κB signaling pathway. This process weakens the inhibitory role of NF-κB in death signaling, leading to the sensitization of cells to cell death signals [46]. On the other hand, another study showed that RNF31 limits caspase-8 activity in complex I and complex II in TRAIL signaling, which causes inhibition of apoptosis [47,48].

## 3. Erythropoiesis and Extrinsic Apoptotic Pathway

### 3.1. Erythropoiesis

In humans, two distinct types of erythroid cell production take place. The first type corresponds to primitive erythropoiesis which occurs in “blood islands” within the yolk sac. The newly formed cells are derived from mesodermal cells. Primitive erythroid cells (EryP) circulating in the early embryo are short-lived (~two days), nucleated, large and express ε-, γ-, α- and ζ -globin genes [10,49]. This process can be regulated by the renal cytokine erythropoietin (Epo). Epo has an influence on the primitive human erythroid precursors’ survival, rate of terminal maturation and proliferation [50]. 

Definitive erythropoiesis occurs first in the fetal liver and then in postnatal bone marrow. The adult erythroid lineage starts with multipotent hematopoietic stem cells (HSCs), also called long-term repopulating HSCs (LT-HSCs). The asymmetrical division of LT-HSCs gives rise to short-term repopulating HSCs (ST-HSCs), the asymmetrical division of which is the source of the multipotent progenitor cells (MPP-HSCs). The difference among these cells’ characteristics is gradual loss of self-renewal potential, but they sustain the same ability to transform into all blood cell types [51]. Further asymmetrical divisions of MPP-HSCs give slowly proliferating BFU-E and then rapidly dividing CFU-E progenitor cells, which divide three to five times within two to three days. Both BFU-E and CFU-E cells are sensitive to Epo due to the presence of erythropoietin receptor (EpoR) on these cells [52,53]. 

Continued stimulation with Epo triggers differentiation into erythroblast precursors: ProE, each of which undergoes three to four rounds of mitosis giving sequentially two basophilic erythroblasts (BasoE), four PolyE, and eight orthochromatic erythroblasts (OrthoE). The maturation processes of these cells are characterized by progressive expansion, accumulation of hemoglobin, decrease in RNA content, increase in chromatin density and decrease in cell size [50]. OrthoE transforms into two cell forms: a pyrenocyte (plasma membrane-coated extruded nucleus, which undergoes phagocytosis by macrophage) and a reticulocyte, which is a stage resulting from enucleation [54] which changes into a mature erythrocyte [8]. Reticulocytes and erythrocytes stop expressing EpoR [55].

Erythropoiesis is regulated by multiple factors such as growth factors and cytokines, SCF, G-CSF, and IL3, vitamins (e.g., folic acid and B12), hormones (corticosteroids, thyroid hormones, activin/inhibin, androgens), iron and its regulators (ferritin, transferrin, TfR-1, TfR-2, ferroportin, hepcidin), survival factors (MCL-1, BCL-xL, HSP70), transcription factors (GATA-1, STAT5A, STAT5B), negative growth regulators (TGFβ, BID, DRs, death ligands), differentiation factors (TGFβ, GDF11 and Epo) [56] and non-coding RNAs (e.g., miRNAs) [57]. Epo plays a crucial role in erythroid maturation. The binding of Epo to its receptor EpoR on the surface of erythroid progenitors causes activation of signal transducer and activator of transcription 5 (Stat5), phosphatidylinositol-3-kinase (PI3K)/Akt and Shc/Ras/mitogen-activated kinase (MAPK) pathways. Inactivation of the Stat5 or PI3K/AKT pathway contributes to significant apoptosis of early progenitors. On the other hand, the loss of the Shc/Ras/MAPK pathway exerts rather slight effects on terminal stages of erythroid maturation [52].

### 3.2. Role of TNF-α in Normal Erythropoiesis

Extensive studies have been devoted to the effects of TNF-α on erythropoiesis. A common feature of these works is that they all show that TNF-α exerted a direct or indirect inhibitory effect on erythroid maturation. One of the first studies showed that the indirect inhibition of human CFU-E colony formation by TNF-α was an effect of interferon β (IFNβ) released by bone marrow accessory cells [58,59]. On the other hand, a direct effect of TNF-α on erythroid progenitors has been described. Namely, TNF-α inhibited erythropoiesis stimulated by Epo alone or in combination with interleukin 3 (IL3) [60,61]. Further study demonstrated that TNF-α can downregulate BFU-E colony formation stimulated by stem cell factor (SCF) with Epo or IL9 with Epo [62]. The same authors showed that TNF-α induced inhibition of BFU-E colony formation is caused by TNFR1/p55. On the other hand, TNF-α receptor 2 (TNFR2/p75) is involved in the inhibition of progenitors’ responses to Epo alone [62,63], which somehow contradicts the fact that TNFR2 does not have a death domain in its cytoplasmic tail [64]. While TNFR1 occurrence was observed only during the initial stages of erythropoiesis, TNFR2 encoding gene (*TNFR2*) expression could be detected during all stages of erythroid differentiation. The variable occurrence of TNFRs on erythroid progenitors may suggest a different sensitivity of these cells to TNF-α [9,65]

Other studies suggested the existence of negative regulatory feedback of TNF-α within specialized niches-erythroblastic islands. The accumulation of mature erythroblasts that express death ligands may temporarily stop the expansion and differentiation of immature erythroblasts sensitive to death ligands. The interaction between death ligands and death receptors resulted in caspase-mediated degradation of GATA-1 [66,67]. A later study suggested the possible occurrence of negative feedback, in which TNF-α secreted by CFU-Es or ProEs suppresses the maturation of erythroid progenitors [68]. Another study carried out on a heterogeneous cell population constituting a hematopoietic stem and progenitor cells (CD34^+^ HSPC) culture demonstrated that TNF-α plays an important role in the weakness in balance between GATA-1 and GATA-2, which is important in erythroid lineage development. TNF-α promoted the formation of the GATA-1/PU.1 complex, which blocks the transcriptional activity of GATA-1, consequently inhibiting erythropoiesis in Epo-induced HSPCs [69]. 

In a dose-dependent manner, TNF-α in combination with interferon γ (INFγ) downregulated growth of purified hematopoietic cells (CD34^+^/CD38^−^ CD34^+^/CD38^+^) in vitro and was also found to induce apoptosis of total bone marrow and CD34^+^ cells [70]. This effect did not require the presence of accessory cells. It seems that TNF-α and INFγ exert a suppressive effect on erythropoiesis in two ways: 1) inhibition of progenitor CD34^+^ cell proliferation by TNF-α and INFγ receptors’ activation-triggered phosphorylation of protein kinase R (PKR) [71] and/or 2) induction of apoptosis, which may cause a reduction in the number of stem and progenitor cells [70]. It should be mentioned that the former mechanism has been implicated in the pathophysiology of bone marrow failure and myelodysplastic (MDS) syndromes [71].

To sum up, it seems that TNF-α mainly exerts an inhibitory effect on erythroid maturation. TNF-α can inhibit erythropoiesis induced by Epo alone, Epo with IL3, Epo with SCF or Epo with IL9. The above-mentioned data indicate that TNF-α acts like negative regulator, which could inhibit maturation of erythroid progenitors and immature erythroblasts or regulate their number via the induction of apoptosis.

### 3.3. Fas/FasL in Normal Erythropoiesis

There is no detectable level of Fas in CD34^+^ cells freshly isolated from human bone marrow. Treatment with TNF-α and/or INFγ increases the Fas level on CD34^+^ cells. The inhibition of erythropoiesis by TNF-α and INFγ might be mediated by the Fas/FasL system [72]. The FasL encoding gene (*FASLG*) expression has not been identified in purified CD34^+^ cells, but there are experimental results which showed that stroma-derived factor 1α (SDF-1α) induced FasL-level increases in these cells with no effect on Fas production. It seems that SDF-1α inhibited the development of erythroid cells by functional upregulation of FasL [73].

The expression of the Fas encoding gene (*FAS*) increases from BFU-E to CFU-E stages of erythroid development, to reach a maximal level in ProE and BasoE. In turn, the *FASLG* gene is expressed in BFU-E, CFU-E and mature OrthoE. The interaction of FasL localized on mature erythroblasts with Fas present on immature erythroblasts causes induction of apoptosis in immature erythroblasts, while mature erythroblasts bearing Fas are insensitive to FasL [74]. Fas–FasL interaction results in apoptotic degradation of the transcription factors Tal-1 and GATA-1. The degradation of both transcription factors is therefore responsible for the Fas-mediated downregulation of maturation of immature erythroblasts [66,75]. This process strongly depends on the concentration of Epo, since Fas-based cytotoxicity against immature erythroblasts could be abrogated by high doses of Epo [74] upregulating GATA-1 expression, which in turn triggers the expression of the *BCL2* gene [76]. At an intermediate level of Epo, cell fate depends on the number of mature erythroblasts in the bone marrow, which means that immature erythroblasts can be arrested in their maturation or enter an apoptosis pathway [77]. 

On the other hand, the suppression of Fas or caspases by siRNA treatment blocked the erythroid differentiation progress at the stage of ProE, i.e., blocking ProE to BasoE transition. This effect was reversed by FasL but not TRAIL treatment and suggests that caspase activation stimulates the erythroid maturation process [78]. 

SCF inhibits activation of caspase-8 and caspase-3 without decreasing the level of Fas. SCF prevents Fas-mediated apoptosis of human erythroid colony-forming cells, mainly CFU-E. This signal was found to depend on Src-family kinase [79,80]. Further studies showed that the mechanism of SCF action is based on the increase of FLIP expression [81]. The results of experiments indicate that a high cellular level of FLIP protects human HSPCs from Fas-mediated apoptosis [82]. 

During erythroid development, various long non-coding RNAs (lncRNAs) were found to regulate erythroid gene expression. Fas-AS1, also known as lncRNA Fas-antisense 1 (Saf), is encoded on the antisense strand of the first intron of the human *FAS* gene on chromosome 10 [83,84]. During erythropoiesis, Fas-AS1 is upregulated by the erythroid transcription factors GATA-1 and Kruppel-like factor 1 (KLF1) and is negatively regulated by NF-κB. Since the level of Fas-AS1 expression increases during erythroid maturation, it is suggested that the role of Fas lncRNA is to protect developing erythroblasts from Fas-mediated apoptosis via reducing the level of Fas [85,86]. 

The studies cited above on the role of the Fas/FasL pathway in erythropoiesis showed that death ligands or receptors are present up to the stage of basophilic erythroblasts. Both are involved in the inhibition of erythropoiesis in CD34^+^ cells and immature erythroblasts. The Fas/FasL system plays a significant role in control of the level and rate of immature erythroblast maturation in an Epo-dependent manner.

### 3.4. Role of TRAIL in Normal Erythropoiesis

Other death receptors expressed by erythroid cells are TRAILR1 and TRAILR2. In immature cells, the expression level of both receptors is higher than in mature erythroblasts [66]. In turn, TRAILR3 and TRAILR4 are not present on the cell surface during erythroid maturation [87]. TRAIL, which binds TRAILR1 and TRAILR2, is produced only by mature erythroblasts [66]. TRAIL, similarly to TNF-α and FasL, negatively regulates adult erythropoiesis [88]. Moreover, TRAIL was found to be involved in INFγ-mediated inhibition of erythropoiesis [89]. 

There is evidence that TRAIL negatively affects generation of mature erythroblasts by activation of the ERK1/2 pathway [87]. Another study showed that protein kinase Cε (PKCε) protects Epo-responsive mature erythroblasts against TRAIL-mediated apoptosis by up-regulation of BCL-2 [90,91]. In addition, recent research showed that in the absence of Epo, TRAIL behaves like a pro-survival factor by activating the NF-κB/IκBα pathway [92]. 

In summary, the TRAIL/TRAILR1 and -R2 system/pathways take part mainly in the negative regulation of erythropoiesis in a similar way as the Fas/FasL pathway or TNF-α. 

### 3.5. Effect of Caspases on Normal Erythroid Maturation

The activation of several caspases seems to be essential in earlier stages of erythroblast differentiation [93]. Proenzymes of caspases 1–3 and 5–9 are present in erythroid cells. The level of procaspases-2, -3, -7 and -8 is the highest in immature erythroblasts [66,94]. Caspase-3 is the best known caspase which is involved in erythroid maturation. The occurrence of activated caspase-3 was observed in cells from late BFU-E [95] to BasoE [93,96]. Caspase-3 inhibition resulted in a reduction in erythroid expansion and differentiation [95]. On the other hand, caspase-3 downregulation had no effect on terminal maturation of erythrocytes, such as nuclear condensation and extrusion [97]. As was mentioned above, caspase activation via the Fas/FasL pathway triggers a positive effect on erythropoiesis; in particular, it is necessary for transition of ProE to BasoE [78]. 

The fate of erythroid precursors was found to depend on the nuclear localization of chaperone HSP70. During erythropoiesis, in the presence of Epo, HSP70 enters the nucleus and protects GATA1 from caspase cleavage. In the absence of Epo, HSP70 leaves the nucleus and allows for the caspase-3-mediated degradation of GATA-1 [98,99].

The above-mentioned data point to the crucial role of caspase-3 in early stages of erythroid maturation and indicate that active caspase-3 is necessary for transition of ProE to BasoE. Moreover, caspase-3 activation plays a major role in apoptosis of erythroid precursors upon Epo starvation.

## 4. Mature Erythrocytes and Extrinsic Apoptotic Pathway

The absence of an intrinsic apoptotic pathway in mature erythrocyte has an obvious evolutionary connection with the lack of mitochondria. Studies on apoptotic machinery in erythrocytes have clearly indicated that caspase-9, apoptotic protease activating factor 1 (Apaf-1) and CytC were missing, but also showed that red blood cells contain considerable amounts of caspase-3 and caspase-8 [100]. Erythrocytes, except for the above-mentioned caspase, also possess Fas, FasL and FADD, which are vital in the extrinsic apoptotic pathway. All of these components are localized to the detergent-resistant membrane (DRM) fraction of aged erythrocytes and also those which have undergone oxidative stress [101].

Early apoptosis activation study attempts showed that erythrocytes do not enter the path of apoptosis after treatment with staurosporine or when cells were incubated in the absence of serum. Normally these conditions trigger apoptosis in nucleated cells [102]. The next studies confirmed that caspase-8 and caspase-3 cannot be activated by CD95-stimulating antibody [103], etoposide or mitomycin c [100], well known inducers of apoptosis. More recent studies revealed that induction of the Fas-mediated cell death cascade may be a result of reactive oxygen species (ROS) and cholesterol accumulation in the erythrocytes of animals exposed to arsenic derivatives (e.g., sodium arsenite) [104,105]. Another study suggested that chronic lead exposure, which increases generation of OH¯ and loss of K^+^ ions, may induce Fas translocation into the membrane DRM fraction and in consequence induction of the extrinsic apoptotic pathway in erythrocytes [106].

It is worth noting that activated caspases can take part in degradation of erythrocyte membrane proteins which are involved in maintenance of cell shape and mechanical properties of the erythrocyte membrane. Caspase-8 is involved in β-spectrin degradation. Experiments performed on erythrocyte ghosts showed that spectrin undergoes proteolytic cleavage among other sites at residue 470, which results in generation of an N-terminal fragment containing the actin binding domain (ABD). This process was shown to depend on protein 4.1 [107]. Activated caspase-3 is able to cleave the N-terminal cytoplasmic domain of human erythroid anion exchanger 1 (AE1/band 3) in aged erythrocytes as a consequence of oxidative stress. This cleavage weakens interaction between AE1 and 4.2 protein, which results in weakened interactions of AE1-based complexes with spectrin [108,109,110,111]. Other studies provided evidence that upon storage of erythrocytes in saline-adenine-glucose-mannitol (SAGM) preservation medium caspase-3 mediates formation of a 24 kDa fragment which is a result of degradation of AE1 protein [112]. Data of other authors showed that caspase-3 is involved in clustering of AE1 protein and recognition of erythrocytes by macrophages [113,114]. Additionally, in erythrocytes, externalization of phosphatidylserine (PS) under oxidative stress is dependent on caspase-3, which seems to be connected to the impairment of flippase activity [115]. 

The observations that mature erythrocytes contain all components of the extrinsic apoptotic pathway raised many questions; however, common inducers of apoptosis do not activate this pathway. Studies based on an animal model suggested that other stimuli, such as ROS or cholesterol accumulation, could activate the extrinsic apoptotic pathway. The above data also demonstrated important effects of caspases-8 and caspase-3 on red cell membrane and membrane skeleton proteins, which are crucial in determining cell shape and erythrocyte deformability and therefore erythrocyte survival in the circulation.

## 5. β-Thalassemia and Extrinsic Apoptotic Pathway

### 5.1. β-Thalassemia

β-thalassemia is an autosomal recessive, inherited blood disorder characterized by reduced (β^+^) or absent (β^0^) beta-globin chain, which is the main component of adult hemoglobin. The beta-globin chain is encoded by the *HBB* gene located on chromosome 11. β-thalassemia is characterized by a decrease in hemoglobin synthesis and reduction of erythrocyte production leading to anemia [116]. β-thalassemia is widespread in the Mediterranean region, Middle East, southeast Asia, and North and Central Africa [117]. Every year, about 68,000 children are born with various β-thalassemia syndromes [118]. The heterogeneity of β-thalassemia is a result of different mutations in the *HBB* gene [119]. 

β-thalassemia minor is caused by heterozygous mutation that affects only one allele of the β-globin gene. This type of thalassemia can be inherited by the β^+^ gene and the β^0^ gene. Carriers of β-thalassemia minor are usually asymptomatic, but sometimes suffer from mild anemia [120]. In turn, β-thalassemia intermedia results from homozygous or compound heterozygous mutations in *HBB* gene, i.e., both alleles are affected. Patients with β-thalassemia intermedia show mild to moderate anemia. The most severe type of β-thalassemia is β-thalassemia major, which occurs when both alleles of the β-globin gene have mutations. Among above types of β-thalassemia, only patients with β-thalassemia major need regular blood transfusions [120,121]. 

Another type of β-thalassemia is β-thalassemia/hemoglobin E, which globally makes up about 50% of all cases of severe β-thalassemia [122]. Hemoglobin E (HbE) is an abnormal hemoglobin which is a result of substitution of a glutamic acid to lysine residue at codon 26 in the *HBB* gene. This mutation contributes to activation of a splice site, which leads to abnormal messenger RNA processing. HbE is produced at a reduced rate and the patient’s symptoms resemble mild β-thalassemia [123]. Patients with β-thalassemia/HbE inherited the β-thalassemia allele from one parent and the structural variant HbE from the other parent [122].

In general, the most important problems in thalassemic patients are iron overload, cardiac arrhythmia, hepatitis, osteoporosis, endocrine disorder and typical symptoms of anemia [119,120,121,122,123,124].

### 5.2. Role of Extrinsic Apoptotic Pathway in Different Types of β-Thalassemia

In 1970, ferrokinetic studies showed that 60–80% of thalassemic erythroid precursors die in the bone marrow. For comparison, in normal cells, this value reaches 10–20% [125]. The β-thalassemic bone marrow contains about six times more erythroid precursors than normal [126]. The cause of ineffective erythropoiesis in a β-thalassemia major patient was shown in 1993. The authors indicated that α-globin chain accumulation may result in accelerated apoptosis of thalassemic erythroid precursors in bone marrow [127]. Quantitative analysis showed that apoptotic cell death in the β-thalassemia major patient was four times higher than in a healthy patient [126]. Analyses carried out on patients with moderate to severe forms of β-thalassemia revealed a correlation between ineffective erythropoiesis and apoptosis. Patients with β-thalassemia/HbE had the most ineffective erythroid maturation and the most accelerated programmed cell death, for which α-globin accumulation was responsible [128]. β-thalassemic major bone marrow contains two times more PolyE and one third fewer OrthoE than normal. In vitro studies identified PolyE as a stage in which apoptosis occurs [129]. The β-thalassemic erythroid precursors are phagocytosed with about twice as high intensity as normal precursors. The enhanced phagocytosis is the result of: 1) an increased level of apoptosis-connected externalization of PS, 2) an increased number of macrophages and their activation [130].

Flow cytometry measurements showed that thalassemic erythroid precursors expose on their surface significantly higher levels of Fas or FasL [131] than in those of normal erythroid cells. As in typical cells, in β-thalassemia binding of FasL to Fas leads to activation of procaspase-8 [132]. Phosphoproteome analysis of β-thalassemic HSCs pointed to very high abundance of FasL, tumor necrosis factor receptor superfamily member 12A (TNFRSF12A) [133] and TRAF2 [123]. The presence and the level of the mentioned proteins may explain why freshly isolated β-thalassemic HSCs were characterized by shorter survival periods than those from normal donors [123,133].

It was observed that in β-thalassemia patients serum levels of TNF-α [134,135,136], IL-1β and IFNγ are higher than in healthy patients [134]. However, recent research suggested that serum levels of TNF-α in thalassemia patients were decreased before and increased after blood transfusion [137]. IL-1β [138,139], TNF-α and IFNγ were found to induce an increased apoptosis level of β-thalassemia/HbE progenitor cells compared to normal progenitors [138]. TNF-α was found to downregulate EpoR encoding gene (*EPOR*) expression and consequently Epo-induced cell proliferation of erythroid progenitor cells in β-thalassemia/HbE patients [136]. 

In contrast to normal erythrocytes, β-thalassemic red blood cells contain a higher level of activated caspase-3, which, similarly to normal cells in a condition of oxidative stress, cleaves AE1 proteins [108,140]. Another example of the results of caspase activation is the cleavage of GATA-1, which becomes unprotected by HSP70 due to its sequestration in the cytoplasm of β-thalassemic erythroblasts by an excess of free α globin chains [141]. Recent data from the research on exportin-1 (XPO1), which is a regulator of HSP70 localization in normal erythroid progenitors, showed the impact of XPO1 activity on β−thalassemic erythroblasts. Namely, XPO1 inhibitor, KPT-251 recovered GATA-1 expression and improved terminal differentiation of β−thalassemic erythroblasts through an increase in the amount of nuclear HSP-70, indicating the possibility of therapeutic treatment of β-thalassemia [142].

It was hypothesized that the excess of ROS could be a reason for increased apoptosis of β-thalassemic erythroid cells. The ROS generation in β-thalassemic precursor cells is probably a result of accumulation of unmatched α-globin, hemichromes, nonheme iron and free iron, the latter being able to generate ROS in a similar way as a Fenton reagent. However, there is no evidence indicating a direct connection between ROS and apoptosis in β-thalassemic erythroid cells [131,143].

The above-mentioned data strongly suggest that ineffective erythropoiesis in β-thalassemia is caused by an increase in the level of apoptosis. In comparison to normal cells, thalassemic cells are characterized by: (1) very high expression of FasL, TNFRSF12A and TRAF2, (2) PolyE-OrthoE arrest, (3) a higher serum level of TNF-α, IL-1β and IFNγ, (4) a higher level of activated caspase-3, and (5) enhanced phagocytosis.

## 6. Conclusions

Erythropoiesis, and mechanisms regulating this process, have attracted the attention of many scientists in the last two decades. One of the key pathways which is involved in the regulation of erythrocyte production is the extrinsic apoptotic pathway, which plays an important role at various stages of erythropoiesis. Moreover, it is interesting to learn the role, if any, of components of the receptor-dependent apoptotic pathway which are present in mature erythrocytes. 

**Figure 4:**
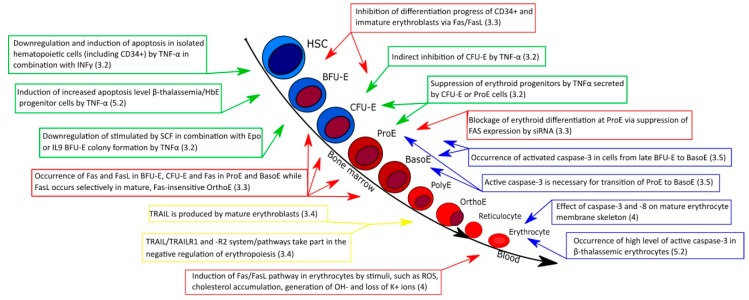
Erythropoiesis and chosen regulatory events connected with apoptotic signals and effectors mentioned in the text. Color code: green: TNFα-, red: Fas/tumor necrosis factor ligand superfamily member 6 (FasL), yellow TRAIL/TNF-related apoptosis-inducing ligand receptor (TRAILR)-, and blue caspase-3/-8-related events.

Understanding all of these processes may help to explain the pathophysiology of anemia associated, among other things, with β-thalassemia. Despite extensive research, it is still unclear what the direct mechanism of apoptosis of erythroid cells in β-thalassemic patients is. It seems necessary to specify the mechanism of accelerated apoptosis induced by α-globin chain accumulation. Additionally, it would be worthwhile to focus on the role of ROS overabundance in the increased apoptosis of thalassemic cells, probably existing as a result of unmatched α-globin accumulation. Furthermore, it would be particularly important to examine antiapoptotic processes occurring in thalassemia patients. Moreover, we should ask the following question: at what erythropoietic stage it is possible to inhibit increased apoptosis in β-thalassemic cells? The answer to this question should facilitate understanding of the main mechanism of accelerated apoptosis in β-thalassemia patients and perhaps aid the development of new therapeutic strategies. The summary of apoptotic processes maintaining regulatory function is presented in Figure 4. It seems that available data [144,145,146] coming from detailed Next Generation Sequencing (NGS) and mass spectrometry-based studies, facilitating transcriptome and proteome analyses at the single cell level, may provide a research background for studies seeking near definitive answers concerning the interplay between regulatory events resulting from the sequential regulation of expression of particular genes and apoptotic signals from other cells and the cellular environment. 

## Abbreviations

BasoE	basophilic erythroblasts
IAP	inhibitors of apoptosis
AMPK	AMP-activated kinase
Apaf-1	apoptotic protease activating factor 1
BAD	BCL2 associated death promoter
BAK	BCL2 antagonist/killer
BAX	BCL2-associated X protein
BCL2	B-cell lymphoma 2
BCLB	BCL-2 like protein 10
BCLW	BCL2-like protein 2
BFL1	BCL2-related protein A1
BFU-E	burst-forming unit-erythroid
BH	BCL2 homology
BID	BH3 interacting-domain death agonist
BIK	BCL2 interacting killer
BIM	BCL2 interacting mediator of cell death
BIR	baculovirus IAP repeat domains
BMF	BCL2 modifying factor
CARD	caspase recruitment domain
c-FLIPs	cellular FLICE (FADD-like IL-1β-converting enzyme) inhibitory proteins
CFU-E	colony-forming unit-erythroid
cIAP1 and cIAP2	cellular inhibitor of apoptosis protein 1 and 2
CRD	cysteine-rich domain
DcRs	decoy receptors
DD	death domain
DED	death effector domain
DISC	death-inducing signaling complex
DRM	detergent-resistant membrane
DRs	death receptors
Epo	erythropoietin
FADD	Fas-associated protein with death domain
Fas	TNF receptor superfamily member 6
FasL	tumor necrosis factor ligand superfamily member 6
HRK	activator of apoptosis harakiri
IAPs	inhibitors of apoptosis
INFγ	interferon γ
JAK3	Janus kinase 3
KLF1	Kruppel-like factor 1
lncRNAs	long non-coding RNAs
LT-HSCs	long-term repopulating HSCs
LUBAC	linear ubiquitin chain assembly complex
MAPK	Shc/Ras/mitogen-activated kinase
MCL1	myeloid cell leukemia
MPCs	multipotent progenitor cells
NGS	Next Generation Sequencing
NF-κB	nuclear factor kappa-light-chain-enhancer of activated B cells
NOXA	Phorbol-12-myristate-13-acetate-induced protein 1
OrthoE	orthochromatic erythroblasts
PAK2	p21-activated kinase 2
PI3K	phosphatidylinositol-3-kinase
PKC	protein kinase C
PKCε	protein kinase Cε
PKR	protein kinase R
PLAD	pre-ligand assembly domain
PolyE	polychromatophilic erythroblasts
ProE	Proerythroblasts
ProEs	Proerythroblasts
PUMA	p53 upregulated modulator of apoptosis
RING	“really interesting new gene” domain
RNF31	ring finger protein 31, also known as HOIP
ROS	reactive oxygen species
SCF	stem cell factor
SDF-1α	stroma-derived factor 1α
Stat5	signal transducer and activator of transcription 5
ST-HSCs	short-term repopulating HSCs
TAB1	TGFβ-activated kinase 1 (TAK1)-binding protein 1
THD	TNF homology domain
TNFR1	TNF-α receptor 1
TNFR2	TNF-α receptor 2
TNFRSF	tumor necrosis factor receptor superfamily
TNFSF	TNF superfamily
TNFα	tumor necrosis factor α
TRADD	TNFR1-associated protein with death domain
TRAF	TNF receptor-associated factor
TRAILR1/TRAILR2	TNF-related apoptosis-inducing ligand receptor 1 and 2
UBA	UB-associated domain
XIAP	X-linked IAP
